# Effect of an Immune-Boosting, Antioxidant and Anti-Inflammatory Food Supplement in Hospitalized COVID-19 Patients: A Prospective Randomized Pilot Study

**DOI:** 10.3390/nu15071736

**Published:** 2023-04-01

**Authors:** Sandra Reino-Gelardo, Marta Palop-Cervera, Nieves Aparisi-Valero, Ignacio Espinosa-San Miguel, Noelia Lozano-Rodríguez, Gonzalo Llop-Furquet, Laura Sanchis-Artero, Ernesto Cortés-Castell, Mercedes Rizo-Baeza, Xavier Cortés-Rizo

**Affiliations:** 1 Internal Medicine Department, Hospital of Sagunto, 46520 Sagunto, Spain; 2 Clinical Analysis and Microbiology Service, Hospital of Sagunto, 46520 Sagunto, Spain; 3 Department of Pharmacology, Pediatrics and Organic Chemistry, Miguel Hernández University, 03690 Alicante, Spain; 4 Department of Nursing, University of Alicante, 03690 Alicante, Spain; 5 Department of Medicine, Cardenal Herrera-CEU University, 46520 Valencia, Spain

**Keywords:** COVID-19, antioxidant, anti-inflammatory, food supplement

## Abstract

Background: COVID-19 disease is a serious global health problem. Few treatments have been shown to reduce mortality and accelerate time to recovery. The aim of this study was to evaluate the potential effect of a food supplement (probiotics, prebiotics, vitamin D, zinc and selenium) in patients admitted with COVID-19. Methods: A prospective randomized non-blinded clinical trial was conducted in a sample of 162 hospitalized patients diagnosed with COVID-19 recruited over eight months. All patients received standard treatment, but the intervention group (*n* = 67) was given one food supplement stick daily during their admission. After collecting the study variables, a statistical analysis was performed comparing the intervention and control groups and a multivariate analysis controlling for variables that could act as confounding factors. Results: ROC curve analysis with an area under the curve (AUC) value of 0.840 (*p* < 0.001; 95%CI: 0.741–0.939) of the food supplement administration vs. recovery indicated good predictive ability. Moreover, the intervention group had a shorter duration of digestive symptoms compared with the control group: 2.6 ± 1.3 vs. 4.3 ± 2.2 days (*p* = 0.001); patients with non-severe disease on chest X-ray had shorter hospital stays: 8.1 ± 3.9 vs. 11.6 ± 7.4 days (*p* = 0.007). Conclusions: In this trial, the administration of a food supplement (Gasteel Plus^®^) was shown to be a protective factor in the group of patients with severe COVID-19 and allowed early recovery from digestive symptoms and a shorter hospital stay in patients with a normal–mild–moderate chest X-ray at admission (ClinicalTrials.gov number, NCT04666116).

## 1. Introduction

The years 2020 and 2021 will be remembered for the emergence of a new virus of the coronavirus family called severe acute respiratory syndrome coronavirus 2 (SARS-CoV-2). SARS-CoV-2, which causes an infection designated by the World Health Organization as Coronavirus Disease 2019 (COVID-19), has spread worldwide, reaching pandemic proportions. The pandemic situation has triggered an unprecedented global health and economic crisis [1]. Repurposing of existing medications has been used widely in studies since the emergence of COVID-19. However, apart from dexamethasone, remdesivir, tocilizumab and baricitinib in selected patients, no medical treatment to date has been shown to improve mortality and/or decrease time to recovery in patients with COVID-19 infection [2,3].

The symptoms caused by COVID-19 disease are nonspecific and may range from asymptomatic to severe pneumonia and death, the most frequent being fever, dry cough, dyspnea, myalgia, fatigue and diarrhea [4]. In the most severe cases, patients may present with pneumonia and acute respiratory distress syndrome (ARDS) and may ultimately die [5]. Among hospitalized patients, the mortality rate is elevated. In the SEMI-COVID national registry (which included more than 15,000 patients throughout Spain through June 2020), a global mortality rate of 21.0% is described, with a marked increase with age (50–59 years: 4.7%; 60–69 years: 10.5%; 70–79 years: 26.9%; ≥80 years: 46.0%) [6].

Since the beginning of the pandemic, it has been observed that a significant percentage of patients present digestive symptoms. These are often the first symptoms of the disease, appearing as early as two days prior to the onset of respiratory symptoms.

Several studies describe the prevalence of gastrointestinal symptoms. In Zhejiang, China, it was noted that of 651 patients with COVID-19, 11.4% had at least one gastrointestinal symptom, with diarrhea being the most frequent [7]. In another study in Hubei of 204 patients, 18.6% had at least one gastrointestinal symptom. It has also been observed that patients with gastrointestinal symptoms are more likely to progress to ARDS and have a greater need for mechanical ventilation or intensive care unit admission (6.8% vs. 2.1%) [8,9]. As the disease becomes more severe, gastrointestinal symptoms are more evident [10]. The presence of active virus in the stool of patients infected by coronavirus has been described [11,12]. The gut microbiota is known to interact with other organs such as the nervous system, the immune system and the respiratory system. An altered gut microbiota increases the risk of alterations in the pulmonary microbiota and has a negative impact on the development of respiratory diseases such as influenza or colds, as well as exacerbations of asthma or COPD. Certain probiotics have been used for the prevention of these diseases. The results have been positive and have demonstrated a possible contribution to the positive evolution of the COVID-19 [13].

In 2005, a randomized controlled clinical trial on the effect of probiotics on the common cold found that the use of probiotics decreased the duration of influenza by almost two days and reduced the severity of respiratory symptoms [14]. A 2015 Cochrane review concluded that there is sufficient scientific evidence that probiotics can support the respiratory tract against colds or flu. It has even been shown that the modulation of the gut microbiota can reduce enteritis and the need for mechanical ventilation associated with pneumonia [15]. The Chinese National Health Commission and the National Administration of Traditional Chinese Medicine recommended probiotics in the treatment of patients with severe COVID-19 infection. In addition, intestinal dysbiosis has been reported in COVID-19 patients, specifically with a decrease in probiotic species of the genera Lactobacillus and Bifidobacterium [16]. The use of probiotics as a preventive food supplement in COVID-19-positive patients and in patients with active COVID-19 infection may therefore have an adjuvant role, with no reported side effects. Vitamin D and elements with zinc and selenium are potent inducers of immune response [17]. Zinc is able to modulate antiviral and antibacterial immunity as well as regulate inflammatory response by affecting both innate and adaptive immunity. Selenium plays a role in anti-inflammatory, antiviral and redox and immune-cell activity. It is useful in both innate and adaptive immunity. Selenoproteins partly reduce oxidative stress generated by viral pathogens. The role of vitamin D in the immune system is well described and is involved on several levels: (1) serves as a physical barrier of epithelial cells in the skin, gut and respiratory system, which protects us from injury or invasion by infection; (2) stimulates the production and secretion of antimicrobial peptides by the intestinal epithelial cells, Paneth cells and intraepithelial lymphocytes in innate immunity; (3) plays a role in innate immunity by modulation of the oxidative burst, promotion of anti-inflammatory cytokines and inhibition of pro-inflammatory cytokines. Low levels of vitamin D are known to increase the risks, severity, morbidity and mortality of several respiratory conditions, such as asthma, tuberculosis, chronic pulmonary disorders, viral respiratory infections and possibly also COVID-19 [17,18].

In this study, we hypothesized that the administration of an antioxidant and anti-inflammatory food supplement could improve the response to disease measured as a shortened hospitalization stay and decreased gastrointestinal symptoms. For this purpose, we used Gasteel Plus^®^, which contains species of the genera described as affected in the digestive microbiome of patients with COVID-19, specifically the strains *Bifidobacterium longum* CECT7347, *Bifidobacterium animalis* subsp. *lactis* CECT8145 and *Lactobacillus rhamnosus* CNCM I-4036. The first two are bifidobacteria and have a strong anti-inflammatory and antioxidant effect. The third strain, CNCM I-4036, has a strong stimulating effect on the immune system. This supplement also contains vitamin D, zinc and selenium. Moreover, in our population, the main predisposing factors for death were established.

## 2. Methods

### 2.1. Trial Oversight

A prospective, randomized, non-blinded clinical trial was carried at Sagunto Hospital, Valencia, Spain. The study was approved by the Clinical Research Ethics Committee of Sagunto Hospital on 31 March 2020 (FXC-30 March 2020) and endorsed by the local ethics committees. The trial was performed in accordance with the principles of the Declaration of Helsinki and registered in the ClinicalTrials.gov registry with the following identifier number: NCT04666116. Heel España S.A.U. laboratories had no influence on the trial design or execution and was not involved in the data collection or analysis, the writing of the manuscript or the decision to submit it for publication. The authors assume responsibility for the accuracy and completeness of the data and analyses, as well as for the fidelity of the trial and this report to the protocol. The trial protocol is available at NEJM.org.

### 2.2. Patients

All participants were COVID-19-RT-PCR-positive subjects who required more than 48 h of hospital admission to the Internal Medicine Service of Sagunto Hospital, Valencia, Spain. Nasopharyngeal samples were collected on the first day of admission for RT-PCR analysis using the VERSANT PCR Molecular System (Siemens, Munich, Germany). Exclusion criteria were age <18 years and hospital discharge in the first 48 h. 

### 2.3. Trial Procedures

Randomization was carried out using the last digit of the patient’s medical record number (MRN). If the MRN ended in an odd number, the food supplement was administered (intervention) and if the MRN ended in an even number, the food supplement was not provided (control). All patients were treated following the treatment protocols of the center’s COVID-19 Committee, which were modified during the inclusion period based on published evidence (detailed information in the Supplementary Materials). The food supplement Gasteel Plus^®^ [19,20,21,22] (Heel España S.A.U. laboratories, Madrid, Spain) is a nutritional supplement containing a mixture of probiotic strains (1:1:1): *Bifidobacterium lactis* BPL1, *Lactobacillus rhamnosus* CNCM I-4036, *Bifidobacterium longum* ES1 and fructooligosaccharides (200 mg) as a prebiotic. Each stick of Gasteel Plus^®^ (300 mg) included lyophilized bacteria powder equivalent to ≥1 × 10^9^ colony-forming units and containing 1.5 mg of zinc, 8.25 μg of selenium, 0.75 μg of vitamin D and maltodextrin as an excipient. The subjects from the intervention group were required to take the sticks once daily during the hospital admission period, preferably in the morning and dissolved in water. The control group was not given a placebo.

For the analysis, the COVID-19 patients were classified according the outcomes to be studied. Mainly, these were mortality, digestive symptoms and radiological involvement on admission by the diagnostic imaging service of Sagunto Hospital following the RALE scale (used for the severity of acute lung edema, differentiating between mild, moderate and severe involvement [23]). The clinical and demographic variables were collected by physicians using the local digitized information system (Integrador, version 6.1) and are provided in Table 1. A summary of the clinical and radiographic characteristics is shown in Table 2. The composition of the treatment administered during hospitalization is found in Tables S1, S3 and S4 and the evolution of the main analytical results is shown in Table S2 in the Supplementary Materials.

As illustrated in Table S2 in the Supplementary Materials, during the recruitment period of this study, we were experiencing the first two “waves” of the pandemic, which allowed us to distinguish differences in the pharmacological treatment of the two groups according to the scientific evidence at the time.

In patients with radiological involvement included in the March–April 2020 period, treatment was mainly based on the use of hydroxychloroquine and azithromycin. In those with radiological worsening, progressive respiratory failure or analytical systemic inflammatory response syndrome criteria, anti-IL6 (Tocilizumab) plus systemic corticosteroids or immunoglobulin treatment was used in cases of bacterial superinfection. In the second stage of recruitment, between September and November 2020, treatment in patients with radiological involvement (mild, moderate or severe) and associated respiratory failure was based on the use of Remdesivir (if less than 7 days of evolution) and dexamethasone 6 mg (if more than 7 days of evolution). In cases of moderate or severe pneumonia with progressive respiratory failure or radiological worsening, we used methylprednisolone bolus (125 mg iv), Tocilizumab (anti-IL6), Anakinra (anti-IL1) or intravenous immunoglobulin, based on the characteristics of the patient. In addition to pharmacological treatment, respiratory support was the other basic pillar in the management of these patients, providing FiO2 from 24% to 100% according to the patient’s needs.

### 2.4. Outcomes

The main outcomes were the assessment of risk factors associated with mortality to determine whether use of the food supplement had a positive effect. These outcomes were (1) reduction in the average duration (days) of persistent digestive symptoms in the patients with gastrointestinal manifestations (measured according to history at admission of at least one of these symptoms: diarrhea, vomiting, abdominal pain or anorexia) and (2) reduction in length of hospital stay (days from admission until discharge) in the patients admitted with COVID-19 infection with normal–mild–moderate chest X-ray severity. Comparisons were made between the group of patients receiving Gasteel Plus^®^ and the control group. 

### 2.5. Statistical Analysis 

According to the GRANMO Sample Size Calculator (version 7.12, April 2012, Barcelona, Spain), accepting an alpha risk of 0.15 and a beta risk of 0.3 in a two-sided contrast, 48 subjects were required in the first group and 48 subjects in the second group to detect statistically significant differences between two proportions, which for group 1 (intervention group) was expected to be a proportion of 0.5 and for group 2 (control group) 0.7 (estimation of 20% of differences). A loss to follow-up rate of 5% was estimated. The arcsine approximation was used.

**Figure 1:**
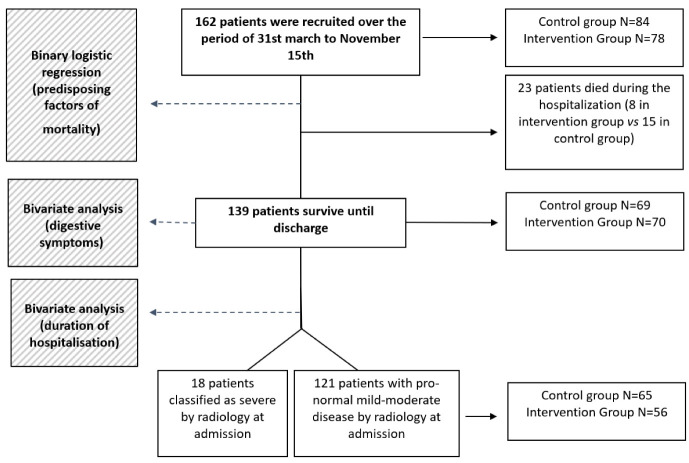
Workflow diagram of the patient recruitment and data analysis scheme.

Quantitative variables are expressed as mean and standard deviation (SD) and student’s *t*-test was calculated assuming a normal distribution based on the central limit theorem. Otherwise, the non-parametric Mann–Whitney U test was used. Qualitative variables are expressed as absolute (*n*) and relative (%) frequencies. The control and intervention groups were compared using the Chi-square test, except in the subgroups in which the sample size was smaller, in which Fisher’s exact was used. First, in the total sample (Figure 1), the possible factors associated with death from COVID-19 (food supplement intake, age, gender, degree of radiological severity at admission and previous disease) were analyzed using binary logistic regression analysis. Next, eliminating the deceased, the days of hospital stay in the control and intervention groups were analyzed using the statistical tests previously described. Prespecified subgroup analyses were performed for the variables shown in Figure 1. Two-sided *p* values of 0.05 or less were considered to indicate statistical significance. The analyses were carried out using the statistical program IBM SPSS, Version 27 (New York, USA). Figure 1 shows the compositions of the cohorts and the statistical approach followed.

## 3. Results

### 3.1. Characteristics of the Patients

From 31 March 2020 to 15 November 2020, a total of 162 patients were enrolled at Sagunto Hospital, 42.0% of whom were women and 58.0% men, with a mean age of 68.7 years. Of these patients, 78 received the food supplement and 84 were assigned to the control group (Figure 1). Treatment was in accordance with the guidelines that were available at the time of the trial (Table S3 in the Supplementary Materials). Of the total number of patients, 23 died during hospitalization (14.2%), 8 in the intervention group (35%) and 15 in the control group (65%). Survivors were classified by radiology at admission in four subgroups: 18 patients presented severe pulmonary involvement on X-ray and 121 patients were classified with mild–moderate disease or no pathogenicity on X-ray (group: normal, mild, moderate and severe). Table 1 and Table 2 show the clinical characteristics among the survival cohorts (*n* = 139, control: 69, intervention: 70). Digestive symptoms (nausea, vomiting, abdominal pain, anorexia or diarrhea) were observed in 37% (52) of patients, 46% of them in the control group and 54% in the experimental group.

The prevalence of hypertension and cardiomyopathy was significantly higher (58% and 31.4%) in the intervention group than in the control group (34.8% and 14.5%) (*p =* 0.01 and *p =* 0.02, respectively) as seen in Table 1. The percentage of patients with dependence for basic activities of daily living in the intervention group was significantly higher (30% vs. 7.2%, *p <* 0.001). A higher number of patients with multiple risk factors were found in the intervention group *(p =* 0.025). Moreover, we found a higher prevalence of underlying disease decompensation in this group (17.1%) than in the control (2.9%), *p* = 0.009. Consequently, analysis of pharmacological treatment usually associated with patient status showed a statistically significant difference only in the use of heparin at an intermediate/full dose (44.3% in the intervention group vs. 24.6%, *p* = 0.02), as shown in Table 2 and Tables S1 and S2 in the Supplementary Materials.

A review of the mean results for blood biochemistry and hematology parameters at admission (Table S2) demonstrated no statistically significant differences between the intervention and control groups

### 3.2. Follow-Up and Outcomes

**Figure 2:**
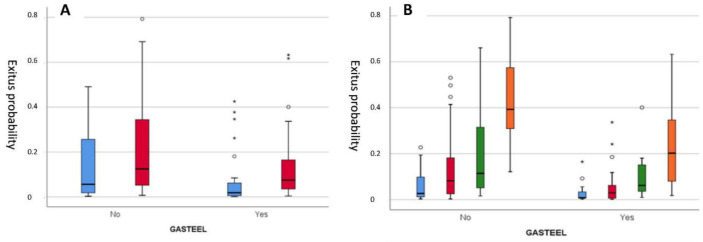
Boxplot of the probability of death calculated using the binary logistic regression model for (**A**) gender (blue: women; red: men) and (**B**) for severity of chest X-ray evaluation (orange = severe, green = moderate, red = mild and blue = no radiological changes) with and without food supplement administration. “°”Values greater than 1.5 times the interquartile range; “*”Values greater than 3 times the interquartile range.

Analysis of the predisposing factors of mortality was the first approach for all 162 patients recruited. A binary logistic regression model for evaluation of survival vs. mortality was carried out. Influencing factors were as follows: (i) female gender (*p* = 0.023; odds ratio (OR) 0.2 (protection); 95%CI: 0.1–0.8); (ii) older age (*p* = 0.001; OR 1.1(for each year); 95%CI: 1.0–1.1) and a higher severity chest X-ray on admission (*p* = 0.001; OR 2.57 (predisposition); 95%CI: 1.4–4.5) (Figure 2). Compared with the control group, the intervention appeared to confer a protective effect, reducing mortality to a level approaching statistical significance (*p* = 0.052, OR 2.9; 95%CI: 1.0–8.5). An ROC (receiver operating characteristic) curve analysis was performed, with an area under the curve (AUC) value of 0.840 (*p* < 0.001; 95%CI: 0.741–0.939), a value that supports a good ability to predict mortality (Figure S1 in the Supplementary Materials). 

Indeed, in the 18 patients with high severity on X-ray (value of 3), a statistically sig- nificant value *p* < 0.001 (Fisher’s test) was found. Death was more frequent in the control group (7/9) compared with the intervention group (1/15).

Digestive symptoms among the survival cohorts were also analyzed, with 52/139 (37.4%) experiencing at least one digestive symptom. Diarrhea was the most common digestive symptom reported in 32/139 (23.0%), followed by anorexia in 28/139 (20.1%), abdominal pain in 18/139 (12.9%) and nausea/vomiting in 15/139 (10.8%). The evolution of gastrointestinal symptoms showed a 1.6 day-reduction in the average period of symptom persistence. We observed an average duration of gastrointestinal symptoms of 4.3 ± 2.2 days (*n* = 25) in the control group versus 2.6 ± 1.3 (*n* = 27) in the intervention group (*p* = 0.001). In addition, after 5 days of hospitalization, 41.2% of the patients in the control group had recovered versus 92.6% in the intervention group (*p* = 0.006). 

**Figure 3:**
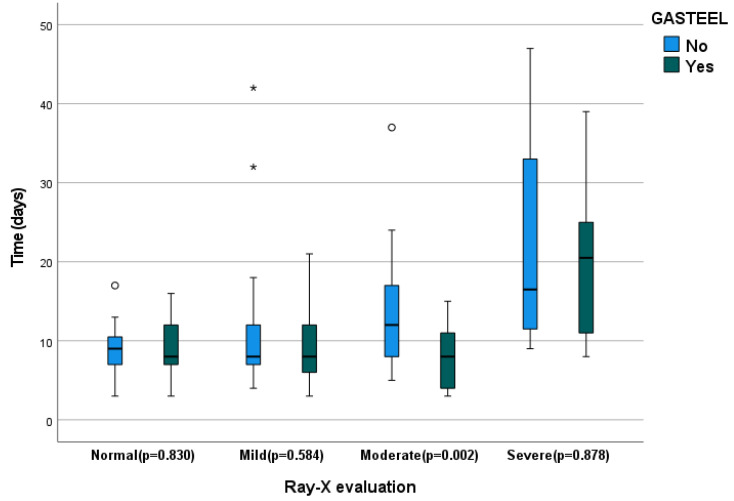
Duration of hospitalization (days) in the patient groups with normal, mild, moderate and severe chest X-ray on admission in the control and intervention groups. “°”Values greater than 1.5 times the interquartile range; “*”Values greater than 3 times the inter-quartile range.

The duration of hospitalization was also analyzed for the 139 surviving patients, but no statistically significant differences were observed (*p* = 0.167). The influence of the level of radiological severity on admission was also studied in each of the subgroups. As shown in Figure 3, the tendency in the high severity subgroup differed from the others. Mainly, the control group had a lower average number of hospitalization days in comparison with the intervention group. Moreover, it was a smaller sample subgroup because there was a large reduction in the number of patients in comparison with the other subgroups due to mortality. However, analysis of each of the subgroups (normal, mild and moderate) revealed statistically significant differences only in the subgroup with moderate radiological severity on admission when comparing the intervention group with the control group (*p* = 0.002). Nevertheless, after exclusion of the subgroup with high severity on chest X-ray, for the new cohort including the expanded normal, mild and moderate groups, the mean hospital stay was 8.7 ± 3.9 days in the intervention group compared with 11.6 ± 7.4 days in the control group. A Student’s t-test showed a statistically significant reduction in hospital stay of 2.9 days for the intervention group compared with the control group (*p* = 0.004). 

## 4. Discussion

As was expected, higher mortality was found in men, older patients and those with severe radiological involvement. Moreover, taking Gasteel Plus^®^ could confer a protective effect with an AUC of 0.84 (*p* < 0.001). Compared to the control group, we observed a reduction in overall mortality. Supporting these results, d’Ettorre et al. [24] described lower mortality in patients taking probiotics compared with the group without probiotic supplementation. In our study, we found a significant reduction in mortality in the subgroup of patients with severe radiological involvement taking Gasteel Plus^®^. However, given the small sample size and the use of immunomodulatory treatments in this subgroup (high-dose corticosteroids, anti-IL1 and anti-IL 6), we believe that protocolized studies with a larger sample size should be performed to be able to assert that Gasteel Plus^®^ has a clinically relevant effect on mortality. 

In our study, diarrhea was the most common digestive symptom (23%). The prevalence of digestive symptoms was slightly higher (37%) than in a recent study by Leal et al. [25], which presented a cohort of 234 Portuguese patients with COVID-19 who required hospitalization from April to March 2020, in which diarrhea (17.9%) was the most common digestive symptom, followed by vomiting (10.9%). The median duration of gastrointestinal symptoms was 9 days (from onset to resolution of gastrointestinal symptoms). The difference compared with our study can be explained by the definition of day 1 of symptoms. We used the day of admission (to measure the effect of our intervention). Digestive symptoms increase the risk of malnutrition and/or dehydration and, therefore, potentially increase the average length of hospital stay and the risk of morbidity and mortality [22]. Tao Zuo et al. [26] and Dhar et al. [10] found significant alterations in COVID-19 fecal microbiomes characterized by the enrichment of opportunistic pathogens. Pan et al. [8] noted that gastrointestinal symptoms are more pronounced as disease worsens and that, indeed, patients are more likely to spend longer periods in the hospital.

A growing body of evidence supports these findings, as the intestinal microbiota can slow the entry of viruses both via the digestive and upper respiratory tracts by strengthening the lung microbiota and thereby preventing or limiting infection [27,28]. There is extensive research investigating the biological roles of the gut microbiota in influencing lung disorders [29]. It is also recognized that viral infections in the respiratory tract cause a disturbance in the gut microbiota [10].

The cytokine storm is an offensive inflammatory response induced by COVID-19, which leads to severe disease in some patients [30]. The search for effective immunomodulatory therapy is consequently one of the main objectives for the treatment of COVID-19. Probiotics have been shown to exert significant effects on strengthening and modulating immune system response against diseases [31,32,33]. They have anti-inflammatory properties during viral infections and contribute to preventing bacterial super-infections. Probiotics also have an immunomodulating role in the cytokine storm (IL-1B, IL-6, IL-15, IL-15, IL-17 IFN-g, TNF-a) [34,35], and it can thus be deduced that probiotics are involved in fighting the cytokine storm associated with COVID-19 [27].

SARS-CoV-2 uses the angiotensin-converting enzyme 2 (ACE2) receptor in human cells as a gateway to penetrate the cell and to replicate. This receptor is expressed in endothelial cells, including intestinal cells, so we should be aware of its possible involvement in the pathophysiology of the disease. Altered activity of this receptor causes impaired expression of antimicrobial peptides in intestinal Paneth cells, which leads to a change in the intestinal microbiota [28]. We can deduce that this influences the gut/lung crosstalk [36]. It is also important to consider that antibiotics and antivirals are often given to patients with COVID-19 infection, which could result in further gut microbiota dysbiosis. Some probiotics belonging to the genus of *Lactobacillus* and *Bifidobacterium* control the gastrointestinal dysbiosis caused by SARS-CoV-2 [37].

In patients with non-severe radiological involvement at admission, the mean length of stay was significantly shorter in the intervention group despite a higher prevalence of risk factors, dependence, and comorbidities (Table 1). Santocrace et al. [28] recommended the use of probiotics and their metabolites to reinforce innate and adaptive immunity in SARS-CoV-2 patients as an adjuvant strategy against complications. Immunomodulatory benefits are particularly relevant for people at risk of developing severe SARS-CoV-2 disease, in whom we find excessive inflammatory responses and complications.

In line with this finding, a study conducted in July 2020 by d’Ettorre et al. [24], which used different probiotic strains as a therapeutic strategy in 28 patients with a confirmed diagnosis of COVID-19, concluded that within 72 h, almost all patients treated showed remission of symptoms, decreased risk of developing respiratory failure, improvement in their clinical conditions and a reduced need for intensive care. Although the sample was small in this analysis, it is plausible that if probiotic administration reduces the average hospital stay and duration of gastrointestinal symptoms, it may also have an effect on clinical improvement in patients with COVID-19-associated pneumonia. These findings strongly correlate with our results in patients with non-severe radiological involvement at admission. 

Despite multiple treatments having been proposed for SARS-CoV-2 infection, the most robust evidence has been found with dexamethasone both in reducing mortality and in the need for mechanical ventilation. Concerning moderate–severe disease, studies with anti-IL-6 receptor monoclonal antibodies (more robust evidence with tocilizumab and baricitinib), remdesivir and anakinra have also described a reduction in mortality and time to clinical recovery. Evidence has recently emerged on treatment in patients with asymptomatic or mild disease with high risk of progression using sotrovimab, remdesivir or nirmatrelvir/ritonavir [38]. The dynamic and changing situation regarding the evidence for different treatments is reflected in the management of our patients during the study period, as can be seen in the Supplementary Materials. Although the treatment administered to our patients was similar in both groups, a significantly greater number of patients were treated with azithromycin in the intervention group. However, given the published evidence on this drug, we do not consider this difference to be clinically relevant.

Vitamin D is known to play an important role in immune function and inflammation, but evidence supporting the efficacy of vitamin D supplementation for the prevention or treatment of COVID-19 is inconclusive [8,39,40]. A systematic review by E. Balboni et al. [41] concluded that there are no studies on the effect of selenium supplementation on COVID-19 infection and the scarce evidence on zinc supplementation does not confirm the efficacy of in vitro studies. Gasteel Plus^®^ contains vitamin D, zinc and selenium. Their beneficial effects could be synergistic with probiotics, but further studies are needed.

As a limitation of the study, because of the situation of overburdened healthcare resources during the COVID-19 emergency, the control group was not given a placebo. To guarantee identical follow-up in both groups, a medical history was taken using the same questionnaire for both groups. Baseline levels of vitamin D, selenium and zinc were also not determined.

## 5. Conclusions

This pilot clinical trial is the first to demonstrate that administration of the food supplement product Gasteel Plus^®^, as an adjuvant to the treatment established in the hospital for SARS-CoV-2-associated pneumonia, reduces the duration of digestive symptoms and hospital stay in patients with mild–moderate pulmonary involvement (determined by chest X-ray). We also observed a non-significant but protective effect of Gasteel Plus^®^ in the high-radiological-severity group. To date, only a limited number of pharmacological interventions have been shown to reduce mortality and average length of stay in patients with COVID-19, demonstrating the importance of this finding using an inexpensive food supplement with no side effects that must be considered in the prevention and treatment of SARS-CoV-2, including severe cases. Further trials with a larger sample size and with a more robust clinical design are necessary to achieve greater external validity and to verify the results of this study.

## Figures and Tables

**Table 1:** Summary of the clinical characteristics of the study survival population. The *p*-values were obtained with the X_2_ test and Student’s *t*-test. Abbreviations: SD, standard deviation; CHF, congestive heart failure; COPD, chronic obstructive pulmonary disease; OSAS, obstructive sleep apnea syndrome; * Risk factor is defined as the presence of at least one comorbidity.

Variable	Intervention Group *n* = 70 Mean ± SD; *n*(%)	Control Group *n* = 69 Mean ± SD; *n*(%)	*p*
Age (years)	70 ± 16	69 ± 17	0.268
Gender (male)	40(57.1)	38(55.1)	0.806
SatO_2_ on admission	94.4 ± 2.5	95.1 ± 3.1	0.160
Days with symptoms	6.9 ± 5.4	7.5 ± 4.0	0.498
Comorbidities:			
- Hypertension	40(58.0)	24(34.8)	0.010
- Obesity	9(13.0)	10(14.9)	0.808
- Diabetes mellitus	11(15.7)	14(20.3)	0.515
- Ischemic Cardiopathy	6(8.6)	4(5.8)	0.745
- Cardiopathy	22(31.4)	10(14.5)	0.026
- CHF	9(12.9)	2(2.9)	0.055
- Asthma	3(4.3)	6(8.7)	0.326
- COPD	6(8.6)	4(5.8)	0.745
- Chronic bronchitis	4(5.7)	0(0.0)	0.120
- OSAS	5(7.1)	4(5.8)	1.00
- Oncological history	6(8.6)	5(7.2)	1.00
- Immunosuppression	3(4.3)	4(5.8)	0.718
- Institutionalized	15(21.4)	8(11.6)	0.170
Dependence for basic activities of daily living	21(30)	5(7.2)	0.001
Clinical decompensation	12(17.1)	2(2.9)	0.009
Smoking	17(56.7)	13(43.3)	0.103
Risk factors (>1)	40(57.1)	22(31.9)	0.024

**Table 2:** Summary of the clinical and radiographic characteristics of the study population at admission. The *p*-values were obtained with the X_2_ test.

Variable	Intervention Group *n* = 70 *n*(%)	Control Group *n* = 69 *n*(%)	*p*
Symptoms on admission			
- Gastrointestinal symptoms	28(40)	24(34.8)	0.600
- Abdominal pain	11(15.7)	7(10.1)	0.450
- Diarrhea	17(24.3)	15(21.7)	0.845
- Fever	41(58.6)	38(55.9)	0.864
- Nausea/vomiting	8(11.4)	7(10.1)	1.000
- Anorexia	12(17.1)	16(23.2)	0.400
Radiography on admission			0.082
- Normal-mild-moderate (0 + 1 + 2)	56(46.3)	65(53.7)	
- Severe (3)	14(20)	4(5.8)	
Need for oxygen support	45(64.3)	45(65.2)	1.000
PaFiO_2_ < 300 at admission	15(21.4)	7(10.1)	0.100

## Data Availability

Not applicable.

## Supplementary Materials

The following supporting information can be downloaded at: https://www.mdpi.com/article/10.3390/nu15071736/s1, Figure S1: ROC curve for evaluation of the potential effect of the food supplement Gasteel as a protector factor in the response. The value of the area under the curve (AUC) indicates the ability of the parameters studied as variable of exitus response; Table S1: Summary of the main treatment administered during hospitalization of the study population, according the local guide. The *p* values were obtained with the χ^2^ test and the Student’s *t* test; Table S2. Evolution of the main analytical parameters for recovery evaluation during admission and discharge period; Table S3. Pharmacological treatment algorithm in the patients recruited from March to April 2020; Table S4. Pharmacological treatment algorithm in the patients recruited September to November 2020. References [42,43,44,45,46,47] are cited in the supplementary materials.
