# Supplementary material for: Effect of an Immune-Boosting, Antioxidant and Anti-Inflammatory Food Supplement in Hospitalized COVID-19 Patients: A Prospective Randomized Pilot Study
Source: Nutrients. 2023 Apr 1;15(7):1736. doi: 10.3390/nu15071736 (PMC10096722; doi:10.3390/nu15071736)
Supplement: Supplementary file 1 [file nutrients-15-01736-s001.zip › nutrients-2295541-supplementary.pdf]

Supplementary Appendix

This appendix has been provided by the authors to give readers additional information about their work.

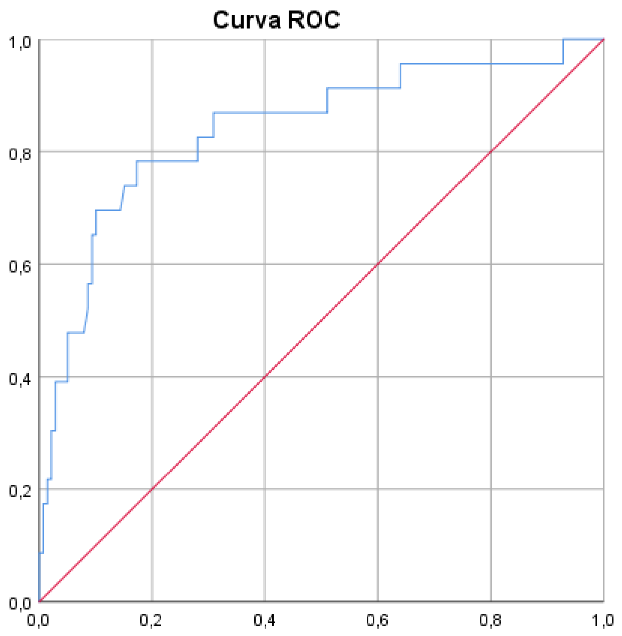

Figure S1.- ROC curve for evaluation of the potential effect of the food supplement Gasteel as a protector factor in the response. The value of the area under the curve (AUC) indicates the ability of the parameters studied as variable of exitus response.

Table S1. Summary of the main treatment administered during hospitalization of the study population, according the local guide. The P values were obtained with the X_2 test and the Student's t test.

Variable	**I**ntervention group **N=70** n **(%)**	**C**ontrol group **N=69** **N(%)**	*p*
Chloroquine	27(38.6)	19(28)	0.209
**MACROLIDES**	65(92.9)	52(75,3)	
Tocilizumab	6(8.6)	2(2.9)	0.275
Corticosteroids:<br>- Dexamethasone<br>- Methylprednisolone	41(58.6)<br>21(30)<br>29(42)	36(52.2)<br>15(21.7)<br>15(21.7)	0.497<br>0.334<br>0.017
Baricitinib	2(2.9)	0(0.0)	0.496
Remdesivir	4(5.8)	1(1.4)	0.366
Anakinra	1(1.4)	0(0.0)	1.00
Inmunoglobulins	1(1.4)	0(0.0)	1.00
Lopinavir/ritonavir	2(2.9)	0(0.0)	0.496
Heparin at prophylactic doses	35(50)	24(34.8)	0.087
Heparin at intermediate / full dose	31(44.3)	17(24.6)	0.020
**ANTIBIOTICS**	30(42.9)	25(36,2)	

Table S2. Evolution of the main analytical parameters for recovery evaluation during admission and discharge period.

Variable	Intervention group n=70 mean±SD;	Control group n=69 mean±SD	*p*
Creatinine at admission (mg/dL)	0.99 ± 0.71	0.91 ± 0.56	0.434
Creatinine at discharge (mg/dL)	0.89±0.41	1.21 ± 2.16	0.237
AST at admission (U/L)	33±30	27±23	0.259
AST at discharge (U/L)	33±34	27±15	0.193
ALT at admission (U/L)	28±28	26±24	0.622
ALT at discharge (U/L)	43±40	38±28	0.388
LDH at admission (U/L)	247±87	251±114	0.817
LDH at discharge (U/L)	225±63	227±94	0.872
Triglycerides at admission (mg/dL)	140±79	144±49	0.719
Triglycerides at discharge (mg/dL)	173±122	166±76	0.757
Ferritine at admission (ng/mL)	721±1067	626±479	0.520
Ferrtine at discharge (ng/mL)	662±614	585±306	0.414
C- Reactive Protein at admission (mg/L)	61±66	55±54	0.524
C- Reactive Protein at discharge (mg/L)	19±31	23±37	0.482
Lymphocyte (absolute value) at admission (10^3/uL)	128±80	122±67	0.639
Lymphocyte (absolute value) at discharge (10^3/uL)	197±395	231±496	0.663
Platelets (absolute value) at admission (10^3/uL)	214±98	221±98	0.642
Platelets absolute value) at discharge (10^3/uL)	250±121	289±116	0.056
Fibrinogen level at admission (mg/dL)	519±173	530±152	0.699
Fibrinogen level at discharge (mg/dL)	398±141	447±169	0.104

Table S3.- Pharmacological treatment algorithm in the patients recruited from March to April 2020.

**No radiological involvment**	**Hydroxychloroquine**
**Mild Pneumonia**	**Azithromycin + Hydroxychloroquine** or<br>Hydroxychloroquine + Lopinavir-ritonavir
**Moderate Pneumonia**	Azithromycin + Hydroxychloroquine or<br>Hydroxychloroquine + Lopinavir-ritonavir
**Severe Pneumonia**	**Azithromycin + Hydroxychloroquine** or<br>Hydroxychloroquine + Lopinavir-ritonavir<br><br>consider adding β1- interferon<br><br>**If clinical, radiological or gasometric progression with analytical criteria of hyperinflammation, consider adding:**<br><br>**Tocilizumab (anti-IL 6) +/-**<br>**Systemic corticosteroids:**<br>- Methylprednisolone 1-2 mg/kg/day during 3– 5 days or<br>- Dexamethasone 20 mg/day during 5 days; in patients with ARDS (acute respiratory distress syndrome) 20 mg/day 5 días following by 10 mg/day five or more days<br>**Intravenous immune globulin therapy if bacterial superinfection**

Table S4.- Pharmacological treatment algorithm in the patients recruited September to November 2020.

**No radiological involvment**	**Symptomatic treatment**
**Mild Pneumonia**	**Remdesivir** if < 7 days from the onset of symptoms plus: peripheral arterial oxygen saturation (SpO_2)$\leq$ 94%, respiratory rate >24 rpm or $PaFiO_2$<300.<br>**Dexamethasone 6 mg** if > 7 days from the onset of symptoms plus SpO_2< 94%.
**Moderate Pneumonia**	**Remdesivir** if < 7 days from the onset of symptoms plus: SpO_2 94%, respiratory rate >24 rpm or $PaFiO_2$<300.<br><br>**Dexamethasone 6 mg** if > 7 days of onset of symptoms plus SpO_2< 94%.<br><br>**Methylprednisolone 125 mg** if absence of clinical, radiological or gasometric improvement in 72 hours of treatment with dexamethasone.
**Severe Pneumonia**	If SpO_2 < 93%, needs of FiO_2 > 0,35 to maintain SpO_2>94%; or PaFiO2 <300:<br>**Remdesivir** if <7 days of clinical and does not require high-flow oxygen therapy / Noninvasive mechanical ventilation<br>**Dexamethasone 6 mg** if >7 days from the onset of symptoms plus SpO_2< 94%.<br><br>**If clinical, radiological or gasometric progression, consider adding:**<br>**Methylprednisolone** (125 or 250 mg).<br>**Tocilizumab (anti-IL6) o Anakinra (anti-IL1).**<br>**Intravenous immune globulin therapy**
**Thromboprophylaxis in all patients**	At prophylactic or intermediate doses in those with a higher risk of thrombosis.

Additional references

1. Anonymous, (2020) Ministerio de Sanidad. Gobierno de España. Documento técnico Manejo clínico del COVID-19: atención hospitalaria 2020. https://www.mscbs.gob.es/profesionales/saludPublica/ccayes/alertasActual/nCov/documentos/Protocolo_manejo_clinico_ah_COVID-19.pdf
2. Anonymous, (2021). World Health Organization. COVID-19 clinical management: living guidance, 25 January 2021. World Health Organization, 2021 https://apps.who.int/iris/handle/10665/338882.

3. Geleris J, Sun Y, Platt J, et al. Observational Study of Hydroxychloroquine in Hospitalized Patients with Covid-19. N Engl J Med. 2020 Jun 18;382(25):2411-2418. doi: 10.1056/NEJMoa2012410.
4. Molina JM, Delaugerre C, Le Goff J, et al. No evidence of rapid antiviral clearance or clinical benefit with the combination of hydroxychloroquine and azithromycin in patients with severe COVID-19 infection. Med Mal Infect. 2020 Jun;50(4):384. doi: 10.1016/j.medmal.2020.03.006
5. Horby P, Lim WS, Emberson JR, et al. Dexamethasone in Hospitalized Patients with Covid-19. N Engl J Med. 2021 Feb 25;384(8):693-704. doi: 10.1056/NEJMoa2021436
6. Kalil AC, Patterson TF, Mehta AK, et al. ACTT-2 Study Group Members. Baricitinib plus Remdesivir for Hospitalized Adults with Covid-19. N. Engl. J. Med. 2021 Mar 4;384(9):795-807. doi: 10.1056/NEJMoa2031994
